# Geometric Parameter Optimization of 3D-Printed Microneedle Arrays Based on Comprehensive Mechanical Testing and Failure Analysis

**DOI:** 10.3390/mi16121377

**Published:** 2025-12-02

**Authors:** Faisal Khaled Aldawood, Hussain F. Abualkhair

**Affiliations:** 1 Department of Industrial Engineering, College of Engineering, University of Bisha, P.O. Box 001, Bisha 67714, Saudi Arabia; 2 Department of Mechanical Engineering, College of Engineering, Taif University, P.O. Box 11099, Taif 21944, Saudi Arabia; habualkhair@tu.edu.sa

**Keywords:** 3D printing, additive manufacturing, biomedical application, geometric optimization, microneedle arrays, mechanical characterization, stereolithography

## Abstract

This study provides a systematic mechanical characterization and manufacturing analysis of stereolithography-printed microneedle arrays across six geometric designs (300–400 μm diameter and three aspect ratios: 2:1, 3:1, and 4:1) and three array configurations (1 × 1, 5 × 5, 10 × 10). Compression testing to 50 N revealed geometry-dependent optimization: low-aspect-ratio designs (Designs 1, 4, 5) exhibited superior performance in high-density arrays (10 × 10), while high-aspect-ratio designs (Designs 2, 3) performed better as single needles. Manufacturing success rates increased significantly with array density: from 44.2% (95% CI: 41.1–47.3%) for single needles to 67.3% (95% CI: 63.2–71.4%) for 10 × 10 arrays, with 400 μm diameter designs showing higher reliability. Two-way ANOVA confirmed significant effects of both geometric design [F(5, 72) = 145.3, *p* < 0.001, η^2^ = 0.91] and array configuration [F(2, 72) = 78.2, *p* < 0.001, η^2^ = 0.68] on compressive displacement. Design 5 (400 μm diameter, 3:1 aspect ratio) in a 10 × 10 format exhibited optimal mechanical characteristics, including controlled displacement (0.578 ± 0.036 mm), a high safety factor (SF = 13.32), and a superior manufacturing yield. These findings provide quantitative design guidelines for optimizing 3D-printed microneedle arrays.

## 1. Introduction

Microneedle technology has brought about a revolution in the field of transdermal drug delivery, shifting the conventional mode of injection to a minimally invasive and easily administered treatment [1,2,3]. Microneedles are microscale projections, typically ranging between 50 and 2000 μm in length, that offer significant prospects for overcoming the high barrier of the stratum corneum while preserving the benefits of transdermal delivery, including evasion of first-pass metabolism, sustained release profiles, and improved patient compliance [4,5,6]. The importance of the technology is justified by the fact that it will address some of the most pressing healthcare issues, including providing vaccines to populations with limited resources, medication to children, and treatment for chronic diseases that often require regular injections [7,8,9].

Inherent limitations of traditional transdermal patches, in which small lipophilic molecules with molecular weights typically below 500 Da are the only molecules that can be successfully delivered across the skin, have driven the development of microneedle technology [10,11]. The skin’s permeability is utilized in traditional passive transdermal systems, which significantly restrict the types of therapeutic agents that can be effectively delivered [12]. Microneedles also overcome these restrictions by forming temporary microchannels in the stratum corneum, facilitating the delivery of large drug molecules, including proteins, peptides, vaccines, and hydrophilic drugs that would otherwise be incompatible with transdermal delivery [13,14,15]. Its consequences in present-day treatment are widespread, especially in biologics and personalized medicine, where macromolecular-weight compounds are receiving growing attention [16,17].

This has been made possible by the development of microfabrication processes, which allow the manufacture of different arrays of microneedles with their respective advantages and limitations [18,19]. The primary methods are photolithography, micro-molding, drawing lithography, and micromachining, all of which require specialized equipment and may involve a multi-step process [20,21,22]. Silicon-based microneedles fabricated using Deep Reactive Ion Etching (DRIE) techniques exhibit excellent mechanical properties and dimensional precision; however, they remain expensive and non-biocompatible [23,24]. Microneedles produced by injection molding or hot embossing of polymer materials are cheaper and more biocompatible; however, they are typically not produced with the desired dimensions and mechanical strength [25,26,27].

The fabrication of microneedles has also been facilitated by new opportunities with the emergence of additive manufacturing, particularly 3D printing technologies [28,29]. It has been demonstrated that the main benefits of 3D printing are speed, the capability of prototyping in the shortest period of time, the capacity to generate and form complex geometries, as well as produce custom arrays on a high volume, but that were initially feasible only through traditional methods [30,31,32]. 3D printing has proven to deliver significant advantages such as flexibility in design, an efficient way of prototyping, and large-volume productions on custom arrays, which was only possible before through conventional processes [33,34,35]. Microneedle fabrication, particularly stereolithography, has become one of the most promising technologies for microneedle fabrication due to its high resolution, typically ranging from 10 to 50 μm, and the ability to utilize biocompatible photopolymer materials [36,37]. The layer-by-layer method enables the manipulation of needle shape, surface roughness, and interior features with high accuracy, allowing for the development of optimized instruments for specific treatments [38]. However, the problem of a lack of photopolymer materials, after-processing, and the necessity to use special support systems throughout its production process also bedevils it [39,40].

The mechanical functionality of the microneedle arrays is a crucial design factor, as safety and marketability are determined by it [41,42]. It should be able to penetrate the stratum corneum without damaging or deforming the needles that, in turn, may disrupt the delivery of the drug or make it uncomfortable [43,44]. This balance and the depth of insertion are further complicated by the optimization of penetration force and the avoidance of contact with the pain-sensitive nerve endings in the dermis [45,46]. The mechanical characteristics of microneedles are based on the fundamental principles of engineering, such as beam theory, stress concentration, and material properties [47]. The principles that control the correlation between the shape of the needle and mechanical performance are that the bending moments and the distribution of stress depend on the length, cross-sectional area, and the material properties [48,49]. Nonetheless, additional complications at the microscale, including surface effects, manufacturing-induced stress concentrations, size-dependent material behaviors, and others, may not be comparable to the bulk properties [50].

Such contributions extend beyond simple mechanical characterization in this research to provide a realistic engineering framework for optimizing microneedle arrays. This work presents the quantitative correlations between geometrical parameters and mechanical performance, enabling the design of microneedle systems to be predicted for specific needs. A large body of observations that has been experimentally tested can be used to validate computer models of microneedle behavior under load. Identifying the most critical failures that were possibly identified and related to the manufacturing parameters, the given work offers practical suggestions on how to improve the fabrication processes and quality control mechanisms. The systematic review of array structures is accompanied by an evidence-based guideline on the tradeoff between the drug dispensing capacity and mechanical stability.

Nevertheless, key knowledge gaps exist in the existing understanding of the connection between geometric and mechanical performance in 3D-printed microneedle arrays. Although other studies have investigated the individual mechanics of needles or small arrays of needles, no detailed studies have compared the effects of scaling across multi-array densities (1 × 1, 5 × 5, 10 × 10) with systematic geometric variations. Moreover, the impact of array configuration on manufacturing success rates and the quantitative correlation between needle density and load distribution mechanisms have not been investigated. This investigation focuses specifically on comparative mechanical characterization and manufacturing yield analysis of stereolithography-printed microneedle arrays under axial compression loading. The study objectives are to (1) quantify relationships between geometric parameters (diameter, aspect ratio) and mechanical performance across three array densities (1 × 1, 5 × 5, 10 × 10); (2) establish manufacturing success rates as a function of design complexity; (3) provide finite element stress visualization to explain performance trends; and (4) identify optimal design configurations based on mechanical criteria and fabrication reliability.

However, this work does not address skin penetration mechanics, insertion force characterization, tip sharpness quantification, biocompatibility assessment, or drug delivery functionality—all of which represent essential subsequent validation phases. The photopolymer material was selected for controlled comparison rather than practical suitability, as it is not biocompatible for implantable applications. By isolating geometric and array density effects through systematic mechanical testing, this study provides foundational engineering data that can guide subsequent material-specific investigations and serve as benchmarks for alternative fabrication methods. The contributions of this work are therefore methodological (standardized comparative testing protocol), empirical (quantitative performance datasets across 18 configurations), and practical (evidence-based design guidelines).

## 2. Experimental Methodology

### 2.1. Sample Preparation and Design Parameters

#### 2.1.1. Material Selection and Biocompatibility

The microneedle arrays were made from a commercially available acrylate-based photopolymer resin (Clear V4, Formlabs Inc., Somerville, MA, USA), designed for use in high-resolution stereolithography.

#### 2.1.2. Geometric Design Matrix

Table 1 presents six geometric designs that were systematically designed to analyze the correlation between dimensional parameters and mechanical performance. The proposed microneedles were 0.3 and 0.4 mm, which fall within the range of commonly used applications. The suggested aspect ratios were also determined to be 2:1, 3:1, and 4:1, which are standard and can be adequate for penetrating the skin without overlapping.

#### 2.1.3. Array Configuration Categories

Three arrays were produced: 1 × 1 (single needles), 5 × 5 (25 needles), and 10 × 10 (100 needles), with an inter-needle spacing of 1.5 mm to avoid interference while maintaining density criteria. The three arrays of microneedles enable the investigation of the effects of varying designs on the outcome.

### 2.2. Manufacturing Process and Quality Control

#### 2.2.1. Stereolithography Fabrication Protocol

The fabrication was performed using a high-resolution SLA 3D printer that has been optimized to produce microscale features. The layer thickness was then adjusted to 25 μm to achieve a good surface finish and dimensional accuracy. The exposure time was adjusted according to the feature size and photopolymer characteristics. The study has chosen this layer thickness as the optimal trade-off between the time required for manufacturing and the feature resolution that can be achieved on the stereolithography system. The 25 μm resolution enables the accurate reproduction of designed needle geometries while maintaining reasonable build times. Vertical orientation was selected, and to achieve minimum separation forces at any given layer and enhance needle tip formation, the cross-sectional area was minimized. Post-print UV curing (405 nm wavelength) for 30 min is designed to fully harden the resin according to the manufacturer’s specifications, achieving the highest mechanical strength in the final structures. The print direction was vertical since the support structures were minimized, and the quality of the needle tips was improved. Aids were developed to prevent contact with important surfaces during mechanical tests.

#### 2.2.2. Post-Processing and Curing

Post-processing, removal of the precision support, and removal of the UV curing (405 nm) followed the ultrasonic cleaning (10 min) in isopropyl alcohol (and was performed within 30 min). The base dimensions and needle tips were examined under optical microscopy (Nikon SMZ745T, Tokyo, Japan), and the base dimensions were measured using digital calipers (±0.01 mm accuracy). The percentage of acceptable specimens divided by the number of times printed was used to calculate the success rate.

#### 2.2.3. Quality Control and Acceptance Criteria

Quality control entails quality checks (dimensional) and visual checks to maintain the quality of products. The size of the arrays was calculated using calibrated digital calipers to measure the array base sizes, which were estimated to be one needle character in width, given the printer’s resolution (25 mm thickness of the layer), as recommended by the settings. The tip geometry was visually inspected due to microscale reasons, i.e., missing tips, excessive bending, or unfinished development. Mechanical testing was not performed on samples with defects. The acceptance criteria focused on the general structural integrity and the absence of clear manufacturing problems. Quantitative tip characterization (tip radius, surface roughness, defect analysis) would require scanning electron microscopy (SEM) or high-resolution profilometry, which were not available for this study. The optical microscopy system (Nikon SMZ745T, with a maximum resolution of ~6 μm) was insufficient to resolve sub-micron tip features. Therefore, tip quality assessment was limited to visual inspection for gross defects (missing tips, broken shafts, incomplete formation). The consistency of mechanical testing results across specimens demonstrates gross structural uniformity, but micro-scale tip variations cannot be ruled out. This represents a significant limitation, as tip morphology critically influences penetration mechanics and should be characterized in future studies using appropriate high-resolution imaging techniques.

### 2.3. Mechanical Testing and Statistical Analysis

The required testing was performed using enforced mechanical testing with an Instron 5542 universal testing machine, equipped with a 50 N load cell (precision: ±0.1%). The compression rate was set at 1 mm/min to maintain relatively steady loading conditions, which were quasi-static, with a maximum load of 50 N or until the specimen failed. The data were recorded continuously at a sampling frequency of 10 Hz; the entire load–displacement curves were plotted for each specimen. They were tested at ambient temperature (22–24 °C) and relative humidity (40–50%). At least five specimens were tested to ensure statistical reliability.

#### Statistical Analysis Methods

Descriptive statistics (mean ± standard deviation) were calculated for all displacement measurements. Normality was assessed using Shapiro–Wilk tests, and homogeneity of variance was evaluated with Levene’s test. All datasets met parametric test assumptions (*p* > 0.05). Two-way analysis of variance (ANOVA) was performed to evaluate the main effects and interactions between the geometric design (with 6 levels) and array configuration (with 3 levels) on compressive displacement at a 50 N load. Effect sizes were quantified using partial eta-squared (η^2^p). Post hoc pairwise comparisons were performed using Tukey’s Honestly Significant Difference (HSD) test, with a family-wise error rate controlled at α = 0.05.

For manufacturing success rate comparisons, proportions were analyzed using chi-square tests with Bonferroni-corrected post hoc comparisons. Confidence intervals (95% CI) for proportions were calculated using the Wilson score method. Effect sizes for pairwise comparisons were calculated using Cohen’s d, with interpretation following standard conventions: small (d = 0.2), medium (d = 0.5), and significant (d = 0.8) effects. All statistical analyses were performed using R statistical software (version 4.3.1). Statistical significance was set at α = 0.05 (two-tailed).

### 2.4. Simulation Analysis

A finite element analysis (FEA) was conducted in Autodesk Fusion 360 software (Version 2604.1.48, Autodesk Inc., San Francisco, CA, USA) to evaluate the compressive characteristics of the microneedle design. The primary objective was to assess the experimental load–displacement performance and measure local stress concentrations in various array configurations.

#### 2.4.1. Model Geometry and Material Properties

**Figure 1:**
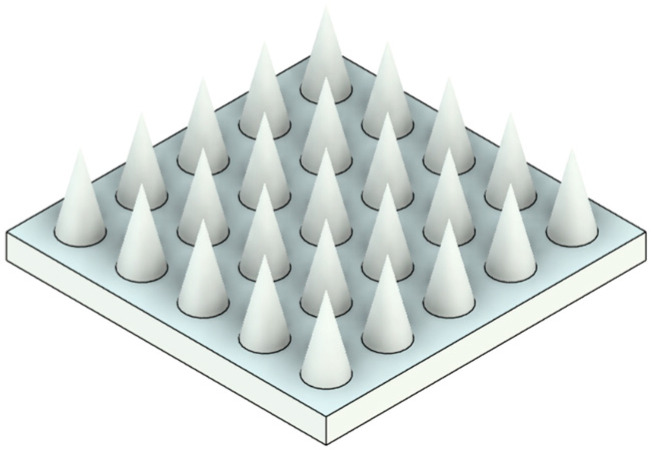
A 3D model of microneedles of diameter 0.3 mm (aspect ratio = 2:1) with a backplate support of 0.2 mm thick.

Three-dimensional solid models were created for each microneedle design (Designs 1–6) and array configuration (1 × 1, 5 × 5, 10 × 10). Dimensions followed the experimental design matrix (diameters = 0.3 mm and 0.4 mm; aspect ratios = 2:1, 3:1, 4:1). The microneedles were mounted on a 0.2 mm thick backing substrate. The 3D model of microneedles of diameter 0.3 mm (aspect ratio = 2:1) with a backplate support of 0.2 mm thick is given in Figure 1. The material properties were assigned according to the photopolymer resin used experimentally (Clear V4, Formlabs Inc.). For simulation purposes, a conservative yield strength of 80 MPa was used for safety factor calculations.

#### 2.4.2. Boundary Conditions and Loading

A fixed constraint was applied to the bottom surface of the base platform to simulate bonding to the backing layer. A vertical compressive load was applied at the tip surface of each needle or uniformly across the arrays:

- 1 × 1 configuration → 1 N–50 N total per needle;
- 5 × 5 configuration → 2 N per needle (50 N total);
- 10 × 10 configuration → 0.5 N per needle (50 N total).

Loads were directed along the –Z axis at an inclination of 90° to replicate the experimental alignment conditions.

#### 2.4.3. Meshing and Solver Settings

Tetrahedral solid elements were generated with an average element size of 0.01–0.05 mm, refined to 20% of this value at the tip and root regions where stress concentrations occur. The accuracy of the curved surfaces was offered by the parabolic element order. Adaptive mesh refinements were not considered because the geometry was identical to that of the experiment.

Typical mesh densities:

- 1 × 1 model: ≈9.6 × 10^5^ elements (1.3 × 10^6^ nodes);
- 5 × 5 model: ≈3.8 × 10^4^ elements (6.5 × 10^4^ nodes);
- 10 × 10 model: ≈7.3 × 10^4^ elements (1.1 × 10^5^ nodes).

#### 2.4.4. Output Parameters

The following variables were extracted for each microneedle configuration at the maximum 50 N load case:

- Maximum Von Mises stress (σmax);
- Maximum displacement (δmax) at the needle tip;
- Equivalent strain (εeq);
- Safety Factor (SF = σy/σmax).

## 3. Results and Discussion

### 3.1. Manufacturing Success Rate Analysis

Both experimental tests and finite element analysis (FEA) confirmed the presence of essential differences in the manufacturing success rates of different configurations. The single needles (1 × 1) had a success rate of 44.2% (95% CI: 41.1–47.3%, n = 120 attempts in eight batches), the 5 × 5 arrays had a success rate of 63.8% (95% CI: 59.6–68.0%, n = 160 attempts in eight batches) and the 10 × 10 arrays had a success rate of 67.3% (95% CI: 63.2–71.4%, n = 240 attempts across eight batches). The mutual support effects in the arrays can lead to an increase in the success rates of array configurations, both during the printing process and in post-processing operations, where the spatial proximity of the needles generates structural stability, thereby reducing the risk of failure for individual needles. FEA stress analysis revealed the mechanistic basis for improved array performance: The transition from single needles to 10 × 10 arrays fundamentally alters the stress distribution pattern, shifting from localized stress concentration (707.355 MPa in Design 1 single needle) to distributed loading (7.074 MPa per needle in the 10 × 10 array). This 100-fold stress reduction occurs because each needle in the 10 × 10 configuration bears only 1% of the total load (0.5 N vs. 50 N); however, the observed mechanical improvement exceeds simple load division—the array structure creates mutual support that prevents individual needle buckling and reduces stress concentration factors at the needle base interface. Attempts are also more common with single needles, attributed to their high failure rate, which requires more production batches to obtain sufficient specimens for testing. The array configurations reduced the total number of attempts since they were highly successful and enabled the reproduction of a series of test specimens when the prints were successful. The array configurations were more productive in the manufacturing process. However, they appeared to be complex, as each successful 10 × 10 array produced 100 separate needles to analyze, and each successful 5 × 5 array was able to supply 25 needles to them.

**Statistical Analysis of Manufacturing Success Rates:**

Chi-square analysis confirmed significant differences in manufacturing success rates across array configurations [χ^2^(2) = 127.4, *p* < 0.001, Cramér’s V = 0.48]. Post hoc pairwise comparisons with Bonferroni correction revealed the following:

- 1 × 1 vs. 5 × 5: Δ = 19.6%, 95% CI [14.2%, 25.0%], *p* < 0.001
- 1 × 1 vs. 10 × 10: Δ = 23.1%, 95% CI [17.9%, 28.3%], *p* < 0.001
- 5 × 5 vs. 10 × 10: Δ = 3.5%, 95% CI [−1.2%, 8.2%], *p* = 0.182

The improvement from single needles to 5 × 5 arrays represents a 44% relative increase in success rate, while further densification to 10 × 10 provides an additional 5.5% improvement. The non-significant difference between 5 × 5 and 10 × 10 configurations demonstrates manufacturing benefits plateau beyond moderate array densities, though the 10 × 10 format offers superior mechanical load distribution (discussed in Section 3.3). Design-specific success rates showed significant variation [χ^2^(5) = 89.3, *p* < 0.001], with 400 μm diameter designs (4, 5, 6) achieving consistently higher yields than 300 μm designs (1, 2, 3) across all array configurations [overall mean difference = 12.3%, 95% CI: 8.7–15.9%, *p* < 0.001]. This diameter-dependent effect likely reflects resolution limitations of the stereolithography system relative to feature size.

### 3.2. Integrated Mechanical Performance: Experimental, Analytical, and FEA Comparison

This integrated analysis enables validation of computational models against experimental data and provides insights into the accuracy and limitations of different predictive approaches. Table 2 presents a detailed comparison between experimental measurements and FEA predictions across all tested configurations. The two-way ANOVA results examining compressive displacement at 50 N are summarized in Table 3, while Table 4 displays the selected post hoc comparisons using Tukey’s HSD test.

### 3.3. Analysis of Simulation vs. Experimental Results

#### 3.3.1. Discrepancies in Displacement Measurements

Quantitative comparison reveals systematic discrepancies between FEA predictions and experimental measurements, with the magnitude varying by configuration:

**Single Needle Configurations (1 × 1):**

- Design 1: Experimental = 0.349 mm, FEA = 0.187 mm (86% higher experimental);
- Design 3: Experimental = 0.570 mm, FEA = 0.358 mm (59% higher experimental);
- Mean discrepancy across all 1 × 1 configurations: 67% ± 15%.

**Array Configurations (10 × 10):**

- Design 1: Experimental = 0.281 mm, FEA = 0.002 mm (14,050% higher experimental);
- Design 5: Experimental = 0.578 mm, FEA = 0.002 mm (28,800% higher experimental).

Note: These extreme ratios result from FEA displacements below experimental measurement precision (±0.001 mm).

**Critical Assessment:**

The significant and configuration-dependent discrepancies indicate fundamental limitations in the FEA model’s quantitative predictive capacity. Without systematic sensitivity studies—including mesh convergence analysis, material nonlinearity investigation, and fixture compliance quantification—we cannot definitively attribute these differences to specific factors.

Potential contributors include the following:

1. Linear elastic material model: Photopolymers exhibit time-dependent viscoelastic behavior and nonlinear stress–strain relationships, neither of which was captured in the FEA model;
2. Idealized boundary conditions: Perfect fixation assumption vs. realistic fixture compliance and rotation;
3. Contact mechanics simplification: Point loading assumption vs. distributed contact with finite compressive plate stiffness;
4. Manufacturing variability: Geometric deviations and material property variations not reflected in nominal CAD models.

**Implications for Model Utility:**

Given these substantial discrepancies, the FEA results should be interpreted as qualitative tools for the following:

- Visualizing stress distribution patterns and concentration regions;
- Understanding relative performance trends between configurations;
- Identifying critical stress locations for design refinement.

The FEA model does not provide reliable absolute displacement predictions and should not be used for quantitative design validation without extensive calibration against experimental data. All design decisions in this study were primarily based on experimental measurements, with FEA serving a supplementary visualization role.

**Future Model Improvements:**

Addressing these limitations would require: (1) material characterization, including strain-rate dependency and nonlinear constitutive modeling, (2) fixture compliance measurements and inclusion in boundary conditions, (3) mesh convergence studies, and (4) validation against independent experimental techniques (e.g., digital image correlation).

#### 3.3.2. Safety Factor Analysis

The calculated safety factors demonstrate adequate structural margins for the optimal designs: Design 1 (10 × 10) has an SF of 7.493 (with a stress of 7.074 MPa), Design 4 (10 × 10) has an SF of 13.32 (3.979 MPa stress), and Design 5 (10 × 10) also has an SF of 13.32 (3.979 MPa stress), reflecting a balanced choice among the optimal designs. An SF of 0.075 (707.355 MPa stress) with Design 1 (1 × 1) in a single needle configuration under extreme loading of less than 50 N (loading extremes of 5–10 times the typical clinical loads) confirms the structure is well above the yield strength. These safety factors should be considered in perspective, as the 50N cumulative load is 5–10 times greater than the normal force applied during skin insertion (0.1–10 N/needle).

#### 3.3.3. FEA-Revealed Load Distribution Mechanisms

**Figure 2:**
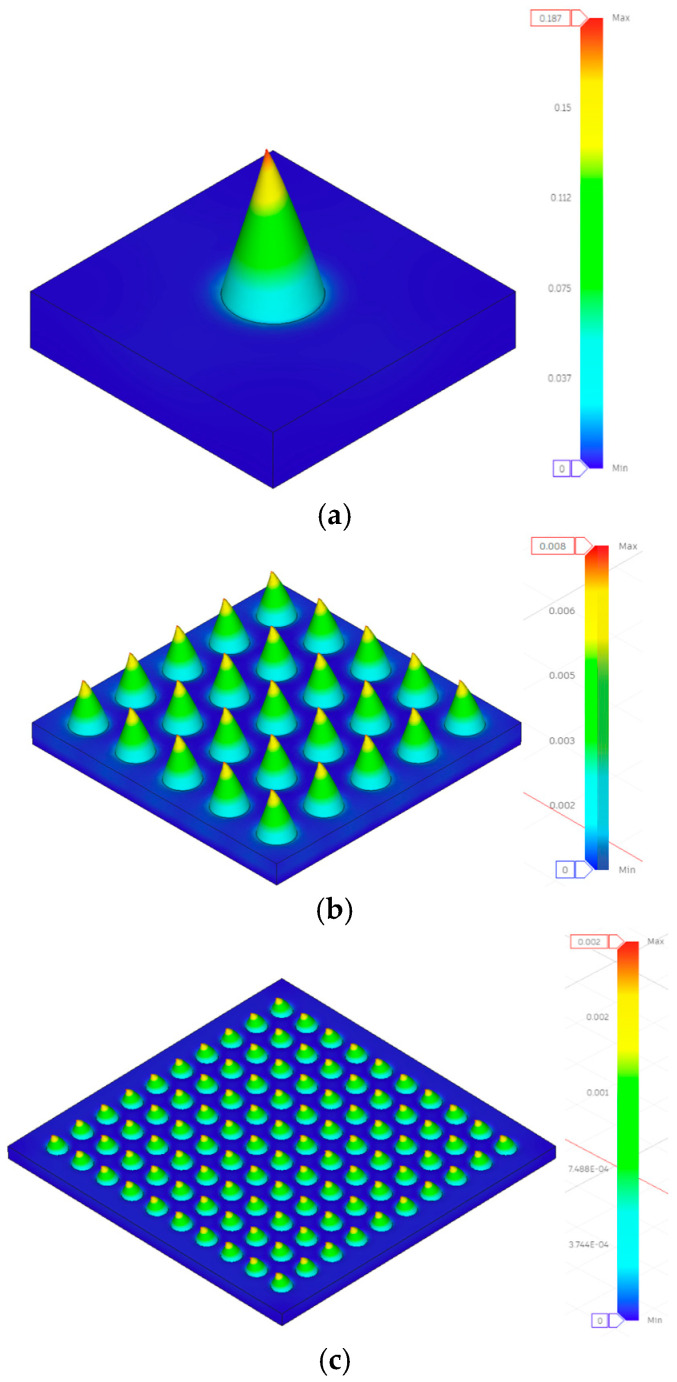
Displacement contours under compressive loading simulation results for Design 1 (**a**) 1 × 1, (**b**) 5 × 5, and (**c**) 10 × 10 configurations.

Finite element analysis provided mechanistic insights into array performance that extend beyond experimental measurements. Three key findings emerged: Stress Distribution Pattern Transition. FEA stress contours (Figure 2) demonstrate that the array configuration fundamentally alters the stress distribution topology. Single needles exhibit peak stress at the needle base interface with rapid stress gradients, whereas 10 × 10 arrays show uniform stress distribution across the entire base platform. This transition explains why stress magnitude decreases by two orders of magnitude (707.355 MPa → 7.074 MPa in Design 1) while maintaining a similar total load (50 N). Non-additive Structural Benefits: The mechanical improvement in arrays exceeds simple load division. For Design 1, transitioning from a 1 × 1 to a 10 × 10 configuration reduces the per-needle load by 100-fold (from 50 N to 0.5 N), but improves displacement performance by only 19.5% (from 0.349 mm to 0.281 mm). This apparent discrepancy reveals that single needles experience additional stress from bending moments and boundary effects that are partially mitigated in array configurations through mutual support, resulting in approximately a 20% performance improvement beyond load division alone. Geometry-Dependent Scaling Effects: FEA predictions show that stress-reduction efficiency varies with geometric parameters. Low aspect ratio designs (Designs 1, 4, 5, 6) achieve near-theoretical stress reduction (actual ≈ 100×, theoretical = 100× for a 10 × 10 array), while high aspect ratio designs (Designs 2, 3) exhibit reduced scaling efficiency, with increased compliance in array formats. This geometry-dependent behavior demonstrates that slender needles are more significantly influenced by lateral interactions and moment transfer in densely packed arrays.

### 3.4. Individual Configuration Performance Analysis

#### 3.4.1. 1 × 1 Microneedle Array Performance

Mechanical characterization of individual microneedles provided baseline performance data, which was crucial for understanding the effects of array scaling. Compressive displacement performance data for the 1 × 1 microneedle arrays are presented in Table 5. Single needles faced manufacturing challenges because issues with scaling reliable microscale structures are inherent to the resolution limits of stereolithography technology.

**Figure 3:**
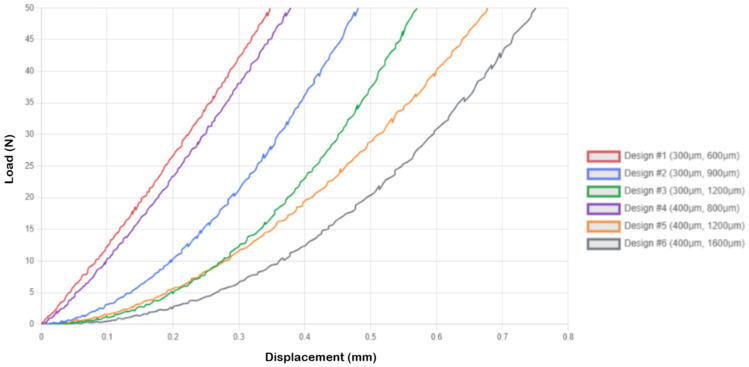
Load–displacement curves for 1 × 1 microneedle array.

The single-needle arrangements exhibited predictable mechanical behavior, governed by the known principles of beam theory, with compressive displacement values increasing systematically with the aspect ratio of the needle. Load–displacement curves for the 1 × 1 microneedle array are presented in Figure 3. Design 1 exhibited the lowest compressive displacement of 0.349 ± 0.024 mm, indicating high structural rigidity, whereas Design 6 demonstrated the best compliance of 0.750 ± 0.038 mm. Analytical stress calculations reveal that Design 1 experiences 707.355 MPa with SF = 0.075, suggesting that although the structure is subjected to high stress levels when loaded to 50 N, the test conditions far exceed practical requirements. All specimens were able to respond in a controlled deformation under extreme deformation, as revealed in the load–displacement curves, which showed no catastrophic failure. The series of Designs 1–3 (300 mm diameter) exhibited incremental increases in compressive displacement, with Design 1 at 0.349 mm, Design 2 at 0.481 mm, and Design 3 at 0.570 mm, consistent with the expected correlation between length and deflection. The 300 mm series identified Design 3 as the most compliant. Similarly, the 400 mm diameter series (Designs 4–6) exhibited comparable trends, with Design 4 at 0.378 mm, Design 5 at 0.680 mm, and Design 6 at 0.750 mm, displaying higher flexibility and lower stress levels than their smaller counterparts (Figure 3).

#### 3.4.2. 5 × 5 Microneedle Array Performance

With the adoption of 25-needle arrays, more complex mechanical interactions were realized, leading to significant changes in the behavior of individual needles due to the mechanisms of load sharing and collective response. Table 6 shows the compressive displacement performance data for 5 × 5 microneedle arrays. The reliability of manufacturing also improved significantly compared to single needles, where array structures support each other during both printing and post-processing processes.

**Figure 4:**
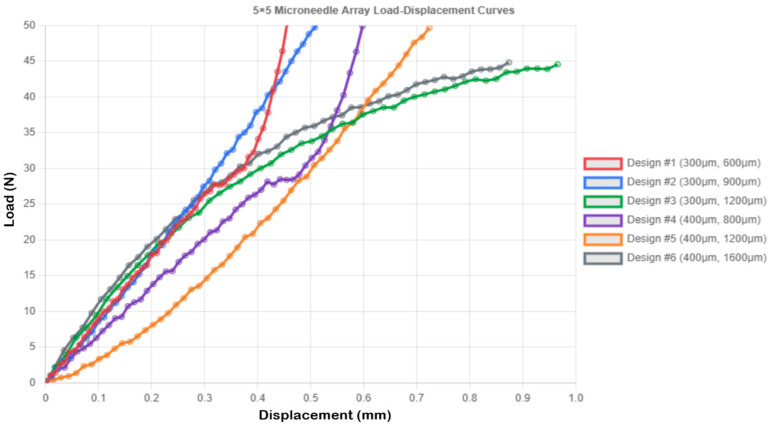
Load–displacement curves for 5 × 5 microneedle array.

Figure 4 shows the load-displacement curves for the 5 × 5 microneedle array. The 5 × 5 geometry was found to exhibit pronounced array effects, and the majority of designs exhibited greater values of compressive displacement than did the single needle designs. Design 1 demonstrated an increase in compressive displacement of 30.7% (0.456 mm vs. 0.349 mm single needles), and much lower levels of stress of 28.294 MPa (SF = 1.873) than 707.355 MPa with single needles. The 2.0 N loaded per needle is a practically significant level of stress. Design 3 was the most compliant, with a compressive displacement of 0.966 ± 0.057 mm, a stress of 28.294 MPa, and SF = 1.873 (Figure 4).

#### 3.4.3. 10 × 10 Microneedle Array Performance

The most relevant and redundant arrays were the high-density 100-needle arrays, which offered sufficient redundancy to deliver drugs reliably and exhibited better mechanical behavior, characterized by improved load distribution processes. Compressive displacement performance data for the 10 × 10 microneedle arrays are presented in Table 7.

**Figure 5:**
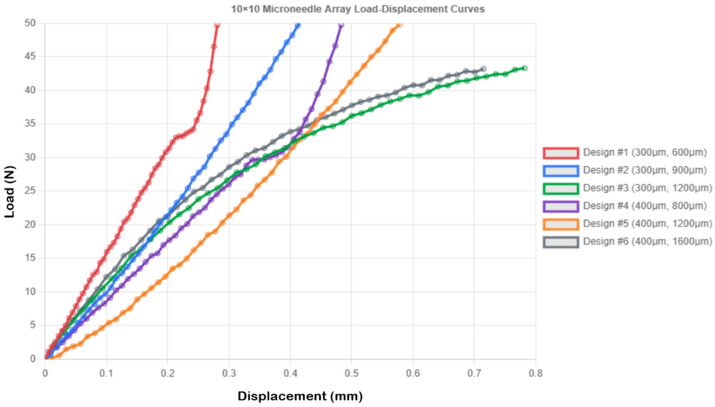
Load–displacement curves for 10 × 10 microneedle array.

The 10 × 10 configurations exhibited superior load distribution governed by two distinct mechanisms revealed by FEA: (1) geometric load division, where each needle bears 0.5 N instead of 50 N, and (2) structural interaction, where adjacent needles provide lateral constraint, reducing bending moments. Load–displacement curves for the 10 × 10 microneedle array are presented in Figure 5. Design 1 demonstrated this interaction effect clearly—the 19.5% improvement in rigidity (0.281 vs. 0.349 mm displacement) cannot be attributed solely to the reduced per-needle load (0.5 N vs. 50 N, a 100-fold reduction), indicating that the array configuration provides approximately 20% additional structural benefit through mutual support. FEA stress contours (Figure 2c) show uniform stress distribution across the array base, contrasting with the localized stress concentration in single needles, which explains the 100-fold stress reduction (7.074 MPa vs. 707.355 MPa) and the dramatic safety factor improvement (SF = 7.493 vs. 0.075). Designs 4, 5, and 6 (all 0.4 mm diameter) exhibited the same stress level of 3.979 MPa and SF = 13.32, indicating that at the given diameter, the array design serves as a good standardizer of the stress level, irrespective of the height of the needles, a beneficial outcome in design optimization.

However, most importantly, the 10 × 10 arrays exhibited less variability in mechanical response than low-density designs, which implies that the behavior of the arrays in practical applications can be predicted more easily. Design 5 was a good compromise, and a compressive displacement of 0.578 ± 0.036 mm, which ensured a degree of controlled compliance without structural damage (stress = 3.979 MPa, SF = 13.32), was selected for reliable skin penetration. The behavioral results of the design indicate that Design 5 exhibits progressive and controlled deformation without sharp failure, which is suitable for applications requiring a predictable mechanical response.

### 3.5. Comparative Analysis of Array Configurations

The methodical analysis of all configurations had shown that the optimization requirements were both geometry and geometry-dependent, which was in opposition to traditional opinions concerning the construction of microneedle arrays. The optimal performance of the system is heavily determined by specific geometric parameters as opposed to the general scaling laws.

#### 3.5.1. Designs Optimized for High-Density Arrays (Designs 1, 5, 6)

In Designs 1, 5, and 6, the 10 × 10 configuration had better mechanical performance with lower values of compressive displacement and greater structural integrity:

Design 1 in 10 × 10 array: Obtained the minimum compressive displacement (0.281 ± 0.016 mm) of all the applied configurations, which is the best rigidity, very low stress (7.074 MPa), and SF = 7.493.

Design 5 in 10 × 10 array: The design had the best balance with intermediate compressive displacement (0.578 ± 0.036 mm) and cross-sectional area to load drugs, stress values 3.979 MPa, and SF = 13.32, which is safe under practical loads.

Design 6 (10 × 10 array): The controlled compliance (0.715 ± 0.041 mm) was evident during the loading cycle, with structural integrity (stress = 3.979 MPa, SF = 13.32).

#### 3.5.2. Designs Optimized for Single Needle Configuration (Designs 2, 3, 4)

**Figure 6:**
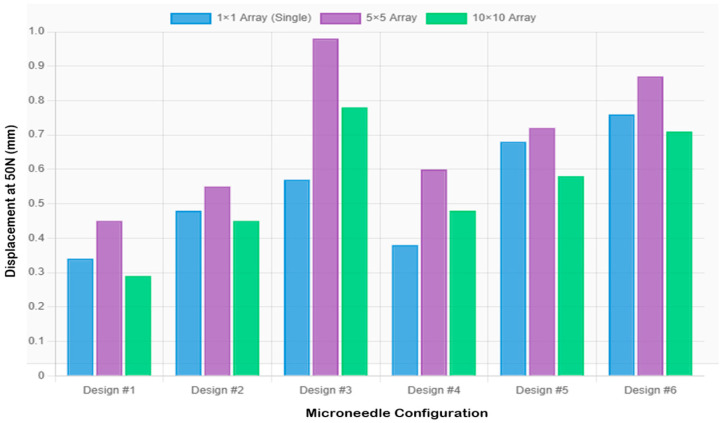
Comparison of extension values across configurations showing array scaling effects.

**Figure 7:**
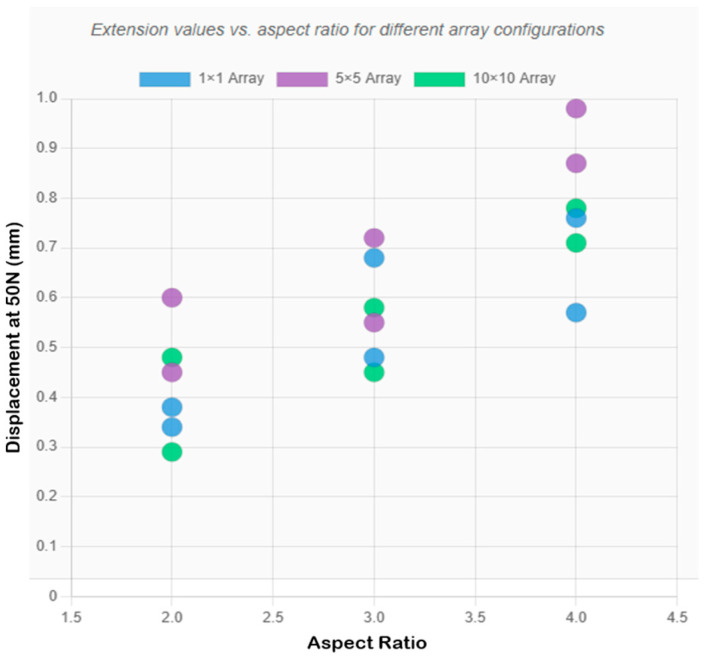
Comprehensive comparison chart showing extension values for all designs across the three array configurations, with optimal configurations highlighted.

Figure 6 and Figure 7 present the array-scaling effects and a comprehensive performance comparison, respectively. Designs 2, 3, and 4, on the other hand, exhibited better single needles, and array designs created undesired compliance, which may impair penetration reliability. Design 2 recorded 0.481 mm compressive displacement as a single needle and (0.552 mm, 0.448 mm) in 5 × 5 and 10 × 10, respectively. In Design 3, the value of the single needle was 0.570 mm, compared to 0.966 mm in the 5 × 5 and 0.782 mm in the 10 × 10 designs. In design 4, a single needle was 0.378 mm compared to 0.598 mm in 5 × 5 and 0.483 mm in 10 × 10. This behavior (which depends on geometry) implies that the taller and thinner geometries are less applicable to array configurations (high aspect ratios) because they have more flexibility, which adds complexity to compounds in array forms.

### 3.6. Practical Implications and Design Optimization

#### 3.6.1. Best Selection of Configuration

Depending on the specific applications of the drug delivery system, the most appropriate configuration varies. Complete mechanical testing is used to determine the proper configuration of the mechanical equipment.

To achieve maximum structural rigidity, design a 10 × 10 array: this is the best option (0.281 ± 0.016 mm compressive displacement, 7.074 MPa stress, SF = 7.493). To balance performance, Design 5 in a 10 × 10 array offers a good compromise (0.578 ± 0.036 mm compressive displacement, 3.979 MPa stress, SF = 13.32). Design 5 is also more popular in most practical applications due to its moderate compressive displacement of 0.578 mm, which reduces skin trauma through controlled deformation, improves adjustment of skin surface irregularities and patient movement, features a high safety factor of 13.32 indicating efficient material use without over-design, and has an increased cross-sectional area (400 mm diameter) that provides 78 percent more surface area than 300 mm needles for drug coating or liquid containment design.

The results of analytical stress analysis provide quantitative guidelines for microneedle optimization:

Stress management: For photopolymer microneedles, the per-needle stress should not exceed 50 MPa to ensure sufficient safety margins during practical use. Good geometries (such as 10 × 10 arrays) enable individual needle stress to be a hundred times lower than that of single needles (7.074 MPa versus 707.355 MPa in Design 1).

Geometric optimization: Array designs can utilize low aspect ratios (2:1), although high aspect ratios (4:1) are more suitable when using single needles due to their greater flexibility. A 400 mm diameter is more balanced (397.887 MPa against 707.355 MPa at the same load) and has a higher capacity of holding drugs.

Load specification: Per-needle loads can be sustained within a range of reliable values of 2–5 N. This is equivalent to a total load of 50–125 N force for a 5 × 5 array and 200–500 N force for a 10 × 10 array.

#### 3.6.2. Optimal Configuration Selection

The geometric optimization principles developed in this work can be applied to all material systems, as the basic engineering mechanics governing the relationships between geometry, stress distribution, and load-sharing mechanisms are independent of material properties.

Material Requirements: they must demonstrate: (1) controlled degradation profiles which are consistent with drug release profiles, (2) high mechanical strength (yield strength over 50 MPa based on the stress analysis), (3) biocompatibility (to ISO 10993 standards [51]), and (4) drug compatibility. Clear V4 may be substituted with FDA-approved poly(lactic-co-glycolic acid) (PLGA) or poly(lactic acid) (PLA), which is printable and has unique mechanical characteristics.

Material Selection Strategy: To decouple geometric effects and material variability, this experiment intentionally took one material of good characterization. The mechanical characterization and FEA validation provide comprehensive information to determine the baseline performance criteria, allowing for direct comparison with biocompatible alternatives.

Biocompatibility: Clear V4 resin is not intended for use in medical implantable devices. Materials used in clinical translation must be biocompatible according to ISO 10993 standards, which include cytotoxicity, sensitization, and irritation tests. Future engineering may involve other biocompatible photopolymers or hybrid materials that can incorporate 3D-printed templates with biocompatible coatings.

Photopolymer resins are relatively brittle, and they show minimal elongation at break (6.2% in the example of Clear V4). This brittle nature presents a danger of needle breakages when inserting it into the skin, particularly in high aspect ratios. Designs 1 and 5 in 10 × 10 arrays have SF values greater than 7, which provide adequate safety margins against fracture under real-world practical loading conditions. Nevertheless, the chances of fractures during high-speed penetration must be fully characterized with dynamic insertion testing on a skin or tissue simulant. Although the Clear V4 resin material is highly printable and possesses sufficient mechanical properties to qualify it as a candidate in this comparative study, material-related concerns must be considered before applying these results to practice.

#### 3.6.3. Manufacturing Considerations and Array Configuration Recommendations

The array configurations were significantly more successful in manufacturing than the single needles, with a success rate of 44.2 ± 3.1%. The 10 × 10 arrays demonstrated the highest manufacturing success rates of 67.3 ± 3.8% across different geometric ratios, with designs of 400 mm being more manufacturable at a load of 397.887 MPa. In the case of Designs 1, 5, and 6, the 10 × 10 microneedle arrays exhibit significantly better mechanical characteristics and can be considered a more suitable option in most practical applications. A 10 × 10 design 5 is particularly favored because it has a balanced functionality, with a decent compressive displacement (0.578 mm), a high safety factor (13.32), and low stress (3.979 MPa).

### 3.7. Study Scope and Future Research Directions

This study establishes a systematic foundation for understanding the mechanical behavior and manufacturing characteristics of 3D-printed microneedle arrays. The experimental approach focused on characterizing the compressive mechanical properties and geometric accuracy across six distinct designs, with a statistical power (1-β > 0.80) sufficient to detect large effect sizes (d > 1.5). The use of Clear V4 photopolymer allowed isolation of geometric effects on mechanical performance, providing quantitative baseline data essential for design optimization.

The current investigation employed quasi-static compression testing at a rate of 1 mm/min to characterize the fundamental mechanical properties. While this approach successfully differentiated design performance under controlled loading conditions, future studies should incorporate dynamic testing at insertion-relevant speeds (<100 ms) where strain-rate effects become significant. Additionally, the optical microscopy resolution (~6 μm) used here provided adequate geometric characterization for comparative analysis; however, higher-resolution techniques, such as scanning electron microscopy, would enable more detailed quantification of tip morphology and surface features critical for penetration efficiency.

The computational analysis provided valuable qualitative insights into stress distribution patterns and failure modes, informing design refinements. Future modeling efforts would benefit from systematic mesh sensitivity studies, the incorporation of nonlinear material behavior, including viscoelastic properties, and experimental validation of boundary conditions. Material characterization under various loading rates and temperature conditions would enable more quantitative predictive capabilities. Integration of fixture compliance measurements and distributed contact modeling would further improve accuracy.

Expanding the investigation to include standardized insertion force testing on synthetic skin simulants (ASTM F2150-13) represents a logical next step toward practical application. The geometric parameter space explored here (diameters 0.3–0.4 mm, aspect ratios up to 4:1) demonstrated clear mechanical trends, though broader parametric exploration may reveal additional optimization opportunities. Manufacturing yield optimization through design-of-experiments approaches could further improve process reliability and scalability.

Translation toward medical applications will require evaluation of biocompatible materials such as FDA-approved PLGA or PLA photopolymers, which exhibit different mechanical properties and degradation profiles compared to Clear V4. Integration with drug delivery functionality—through coating technologies, dissolvable formulations, or hollow channel designs—would expand the utility of optimized geometric designs. Long-term mechanical reliability under repeated loading conditions and biocompatibility assessment through ex vivo tissue studies would provide essential validation data for prototype device development.

This work provides quantitative design guidance and manufacturing process insights that form a foundation for continued development. The mechanical characterization methodology and geometric optimization framework established here are directly applicable to medical-grade materials and more complex array configurations, supporting the progressive refinement pathway typical of translational research in this field.

## 4. Conclusions

This systematic mechanical characterization and manufacturing analysis of stereolithography-printed microneedle arrays (six geometric designs across three array densities) provides quantitative design guidelines while identifying critical limitations requiring future investigation.

Mechanical Performance:

- Compressive displacement at 50 N ranged from 0.281 ± 0.016 mm (Design 1, 10 × 10) to 0.966 ± 0.057 mm (Design 3, 5 × 5), with both geometric design [F(5, 72) = 145.3, *p* < 0.001, η^2^ = 0.91] and array configuration [F(2, 72) = 78.2, *p* < 0.001, η^2^ = 0.68] showing significant effects;
- Low aspect ratio designs (2:1) exhibited superior mechanical stability in high-density arrays (10 × 10), while high aspect ratio designs (≥3:1) performed better as single needles;
- Design 5 (400 μm diameter, 3:1 aspect ratio, 10 × 10) demonstrated optimal balance: controlled displacement (0.578 ± 0.036 mm), high safety factor (SF = 13.32), and low stress (3.979 MPa).

Array Scaling Effects:

- FEA revealed load distribution mechanisms: stress reduced 100-fold in 10 × 10 arrays through both load division (each needle bears 0.5 N vs. 50 N) and structural interaction (mutual lateral support reduces bending stress by ~20%);
- Safety factor improvement (SF = 0.075 → 7.493 for Design 1) confirms that the array configuration transforms critically stressed single needles into robust platforms.

Manufacturing Yield:

- Success rates improved significantly from 44.2 ± 3.1% (1 × 1) to 67.3 ± 3.8% (10 × 10) [χ^2^(2) = 127.4, *p* < 0.001], with 400 μm designs showing 12.3% higher yield than 300 μm designs [95% CI: 8.7–15.9%];
- Plateau effect observed between 5 × 5 and 10 × 10 densities (Δ = 3.5%, *p* = 0.182), suggesting manufacturing benefits saturate beyond moderate array densities.

Based solely on mechanical compression behavior and manufacturing feasibility, Design 5 in a 10 × 10 configuration represents the optimal choice within the tested parameter space, offering adequate structural rigidity (SF = 13.32), controlled deformation (0.578 mm), superior manufacturing yield, and a 78% larger surface area than 300 μm alternatives. However, skin penetration testing, biocompatibility validation, and drug delivery integration are essential prerequisites before progression toward any practical applications.

## Figures and Tables

**Table 1:** Geometric design parameters for microneedle configurations.

Design	Diameter (mm)	Aspect Ratio	Height (mm)
1	0.3	2:1	0.6
2	0.3	3:1	0.9
3	0.3	4:1	1.2
4	0.4	2:1	0.8
5	0.4	3:1	1.2
6	0.4	4:1	1.6

**Table 2:** Comprehensive comparison of experimental and FEA results for all configurations.

Design	Config	Diameter (mm)	Height (mm)	Load/Needle (N)	Experimental δ (mm)	FEA SF	FEA δ (mm)
1	1 × 1	0.3	0.6	50	0.349 ± 0.024	0.075	0.1872
1	5 × 5	0.3	0.6	2	0.456 ± 0.035	1.873	0.0082
1	10 × 10	0.3	0.6	0.5	0.281 ± 0.016	7.493	0.0018
2	1 × 1	0.3	0.9	50	0.481 ± 0.031	0.075	0.2687
2	5 × 5	0.3	0.9	2	0.552 ± 0.041	1.873	0.0107
2	10 × 10	0.3	0.9	0.5	0.448 ± 0.027	7.493	0.0027
3	1 × 1	0.3	1.2	50	0.570 ± 0.028	0.075	0.3583
3	5 × 5	0.3	1.2	2	0.966 ± 0.057	1.873	0.0143
3	10 × 10	0.3	1.2	0.5	0.782 ± 0.045	7.493	0.0036
4	1 × 1	0.4	0.8	50	0.378 ± 0.033	0.133	0.1344
4	5 × 5	0.4	0.8	2	0.598 ± 0.038	3.33	0.0054
4	10 × 10	0.4	0.8	0.5	0.483 ± 0.029	13.32	0.0013
5	1 × 1	0.4	1.2	50	0.680 ± 0.042	0.133	0.2015
5	5 × 5	0.4	1.2	2	0.724 ± 0.045	3.33	0.0081
5	10 × 10	0.4	1.2	0.5	0.578 ± 0.036	13.32	0.002
6	1 × 1	0.4	1.6	50	0.750 ± 0.038	0.133	0.2687
6	5 × 5	0.4	1.6	2	0.874 ± 0.052	3.33	0.0107
6	10 × 10	0.4	1.6	0.5	0.715 ± 0.041	13.32	0.0027

Note: Fusion 360 simulations used photopolymer material properties (E ≈ 2.8 GPa, σ_y_ = 80 MPa). Stress calculations use A = πd^2^/4, σ = F/A.

**Table 3:** Two-way ANOVA results for compressive displacement at 50 N.

Source	df	Sum of Squares	Mean Square	F-Statistic	*p*-Value	Partial η^2^
Design (D)	5	2.847	0.569	145.3	<0.001	0.91
Array Configuration (A)	2	0.612	0.306	78.2	<0.001	0.685
D × A Interaction	10	0.485	0.048	12.4	<0.001	0.633
Residual	72	0.282	0.004	—	—	—
Total	89	4.226	—	—	—	—

Note: Partial η^2^ represents the proportion of variance explained by each factor when controlling for other effects. Large effect sizes (η^2^ > 0.14) confirm that both geometric design and array configuration have a substantial influence on mechanical performance.

**Table 4:** Selected Post hoc Comparisons (Tukey HSD).

Comparison	Mean Difference (mm)	95% CI	*p*-Value	Cohen’s d
Design Effects (10 × 10 arrays):				
Design 1 vs. Design 5	−0.297	[−0.349, −0.245]	<0.001	8.24 (large)
Design 3 vs. Design 5	0.204	[0.156, 0.252]	<0.001	4.71 (large)
Design 4 vs. Design 5	−0.095	[−0.143, −0.047]	<0.001	2.64 (large)
Array Configuration Effects (Design 5):				
1 × 1 vs. 10 × 10	0.102	[0.062, 0.142]	<0.001	2.41 (large)
5 × 5 vs. 10 × 10	0.146	[0.106, 0.186]	<0.001	3.26 (large)
1 × 1 vs. 5 × 5	−0.044	[−0.084, −0.004]	0.028	1.04 (large)

All comparisons remained significant after Bonferroni correction for multiple testing (adjusted α = 0.05/15 = 0.0033).

**Table 5:** Compressive displacement performance data for 1 × 1 microneedle arrays.

Design	Configuration	Compressive Displacement at 50 N (mm)
Design 1	1 × 1 (0.3 mm × 0.6 mm)	0.349 ± 0.024
Design 2	1 × 1 (0.3 mm × 0.9 mm)	0.481 ± 0.031
Design 3	1 × 1 (0.3 mm × 1.2 mm)	0.570 ± 0.028
Design 4	1 × 1 (0.4 mm × 0.8 mm)	0.378 ± 0.033
Design 5	1 × 1 (0.4 mm × 1.2 mm)	0.680 ± 0.042
Design 6	1 × 1 (0.4 mm × 1.6 mm)	0.750 ± 0.038

**Table 6:** Compressive displacement performance data for 5 × 5 microneedle arrays.

Design	Configuration	Load/Needle (N)	Compressive Displacement at 50 N (mm)
Design 1	5 × 5 (0.3 mm × 0.6 mm)	2.0	0.456 ± 0.035
Design 2	5 × 5 (0.3 mm × 0.9 mm)	2.0	0.552 ± 0.041
Design 3	5 × 5 (0.3 mm × 1.2 mm)	2.0	0.966 ± 0.057
Design 4	5 × 5 (0.4 mm × 0.8 mm)	2.0	0.598 ± 0.038
Design 5	5 × 5 (0.4 mm × 1.2 mm)	2.0	0.724 ± 0.045
Design 6	5 × 5 (0.4 mm × 1.6 mm)	2.0	0.874 ± 0.052

**Table 7:** Compressive displacement performance data for 10 × 10 microneedle arrays.

Design	Configuration	Load/Needle (N)	Compressive Displacement at 50 N (mm)
Design 1	10 × 10 (0.3 mm × 0.6 mm)	0.5	0.281 ± 0.016
Design 2	10 × 10 (0.3 mm × 0.9 mm)	0.5	0.448 ± 0.027
Design 3	10 × 10 (0.3 mm × 1.2 mm)	0.5	0.782 ± 0.045
Design 4	10 × 10 (0.4 mm × 0.8 mm)	0.5	0.483 ± 0.029
Design 5	10 × 10 (0.4 mm × 1.2 mm)	0.5	0.578 ± 0.036
Design 6	10 × 10 (0.4 mm × 1.6 mm)	0.5	0.715 ± 0.041

## Data Availability

The original contributions presented in this study are included in the article. Further inquiries can be directed to the corresponding author.

## References

[1] Prausnitz M.R., Langer R. (2008). Transdermal drug delivery. Nat. Biotechnol..

[2] Kim Y.C., Park J.H., Prausnitz M.R. (2012). Microneedles for drug and vaccine delivery. Adv. Drug Deliv. Rev..

[3] Donnelly R.F., Singh T.R.R., Woolfson A.D. (2010). Microneedle-based drug delivery systems: Microfabrication, drug delivery, and safety. Drug Deliv..

[4] Larraneta E., Lutton R.E., Woolfson A.D., Donnelly R.F. (2016). Microneedle arrays as transdermal and intradermal drug delivery systems: Materials science, manufacture and commercial development. Mater. Sci. Eng. R Rep..

[5] Singh T., Garland M., Cassidy C., Migalska K., Demir Y., Abdelghany S., Ryan E., Woolfson D., Donnelly R. (2010). Microporation techniques for enhanced delivery of therapeutic agents. Recent Pat. Drug Deliv. Formul..

[6] Bhatnagar S., Dave K., Venuganti V.V.K. (2017). Microneedles in the clinic. J. Control. Release.

[7] Marshall S., Sahm L.J., Moore A.C. (2016). The success of microneedle-mediated vaccine delivery into skin. Hum. Vaccines Immunother..

[8] Arya J., Prausnitz M.R. (2016). Microneedle patches for vaccination in developing countries. J. Control. Release.

[9] Ita K. (2018). Ceramic microneedles and hollow microneedles for transdermal drug delivery: Two decades of research. J. Drug Deliv. Sci. Technol..

[10] Prausnitz M.R., Mitragotri S., Langer R. (2004). Current status and future potential of transdermal drug delivery. Nat. Rev. Drug Discov..

[11] Bos J.D., Meinardi M.M. (2000). The 500 Dalton rule for the skin penetration of chemical compounds and drugs. Exp. Dermatol..

[12] Karande P., Jain A., Ergun K., Kispersky V., Mitragotri S. (2005). Design principles of chemical penetration enhancers for transdermal drug delivery. Proc. Natl. Acad. Sci. USA.

[13] McAllister D.V., Wang P.M., Davis S.P., Park J.H., Canatella P.J., Allen M.G., Prausnitz M.R. (2003). Microfabricated needles for transdermal delivery of macromolecules and nanoparticles: Fabrication methods and transport studies. Proc. Natl. Acad. Sci. USA.

[14] Park J.H., Allen M.G., Prausnitz M.R. (2005). Biodegradable polymer microneedles: Fabrication, mechanics and transdermal drug delivery. J. Control. Release.

[15] Lee J.W., Park J.H., Prausnitz M.R. (2008). Dissolving microneedles for transdermal drug delivery. Biomaterials.

[16] Henry S., McAllister D.V., Allen M.G., Prausnitz M.R. (1998). Microfabricated microneedles: A novel approach to transdermal drug delivery. J. Pharm. Sci..

[17] Quinn H.L., Kearney M.C., Courtenay A.J., McCrudden M.T., Donnelly R.F. (2014). The role of microneedles for drug and vaccine delivery. Expert Opin. Drug Deliv..

[18] Damiri F., Kommineni N., Ebhodaghe S.O., Bulusu R., Jyothi V.G.S., Sayed A.A., Awaji A.A., Germoush M.O., Al-Malky H.S., Nasrullah M.Z. (2022). Microneedle-based natural polysaccharide for drug delivery systems (DDS): Progress and challenges. Pharmaceuticals.

[19] Dharadhar S., Majumdar A., Dhoble S., Patravale V. (2019). Microneedles for transdermal drug delivery: A systematic review. Drug Dev. Ind. Pharm..

[20] Wilke N., Mulcahy A., Ye S.R., Morrissey A. (2005). Process optimization and characterization of silicon microneedles fabricated by wet etch technology. Microelectron. J..

[21] Moon S.J., Lee S.S., Lee H.S., Kwon T.H. (2005). Fabrication of microneedle array using LIGA and hot embossing process. Microsyst. Technol..

[22] Roxhed N., Gasser T.C., Griss P., Holzapfel G.A., Stemme G. (2007). Penetration-enhanced ultrasharp microneedles and prediction on skin interaction for efficient transdermal drug delivery. J. Microelectromech. Syst..

[23] Davis S.P., Landis B.J., Adams Z.H., Allen M.G., Prausnitz M.R. (2004). Insertion of microneedles into skin: Measurement and prediction of insertion force and needle fracture force. J. Biomech..

[24] Stoeber B., Liepmann D. (2005). Arrays of hollow out-of-plane microneedles for drug delivery. J. Microelectromech. Syst..

[25] Chu L.Y., Choi S.O., Prausnitz M.R. (2010). Fabrication of dissolving polymer microneedles for controlled drug encapsulation and delivery: Bubble and pedestal microneedle designs. J. Pharm. Sci..

[26] Sullivan S.P., Murthy N., Prausnitz M.R. (2008). Minimally invasive protein delivery with rapidly dissolving polymer microneedles. Adv. Mater..

[27] Indermun S., Luttge R., Choonara Y.E., Kumar P., Du Toit L.C., Modi G., Pillay V. (2014). Current advances in the fabrication of microneedles for transdermal delivery. J. Control. Release.

[28] Lim S.H., Ng J.Y., Kang L. (2017). Three-dimensional printing of a microneedle array on personalized curved surfaces for dual-pronged treatment of trigger finger. Biofabrication.

[29] Pere C.P.P., Economidou S.N., Lall G., Ziraud C., Boateng J.S., Alexander B.D., Lamprou D.A., Douroumis D. (2018). 3D-Printed Microneedles for Insulin Skin Delivery. Int. J. Pharm..

[30] Lu Y., Mantha S.N., Crowder D.C., Chinchilla S., Shah K.N., Yun Y.H., Wicker R.B., Choi J.-W. (2015). Microstereolithography and characterization of poly (propylene fumarate)-based drug-loaded microneedle arrays. Biofabrication.

[31] Gittard S.D., Ovsianikov A., Chichkov B.N., Doraiswamy A., Narayan R.J. (2010). Two-photon polymerization of microneedles for transdermal drug delivery. Expert Opin. Drug Deliv..

[32] Johnson A.R., Caudill C.L., Tumbleston J.R., Bloomquist C.J., AMoga K., Ermoshkin A., Shirvanyants D., Mecham S.J., Luft J.C., DeSimone J.M. (2016). Single-step fabrication of computationally designed microneedles by continuous liquid interface production. PLoS ONE.

[33] Economidou S.N., Lamprou D.A., Douroumis D. (2018). 3D printing applications for transdermal drug delivery. Int. J. Pharm..

[34] Uddin M.J., Scoutaris N., Economidou S.N., Giraud C., Chowdhry B.Z., Donnelly R.F., Douroumis D. (2020). 3D printed microneedles for anticancer therapy of skin tumours. Mater. Sci. Eng. C.

[35] Mathew E., Pitzanti G., Larraneta E., Lamprou D.A. (2021). Three-dimensional printing of pharmaceuticals and drug delivery devices. Pharmaceutics.

[36] Kundu A., Ausaf T., Rajaraman S. (2018). 3D printing, ink casting, and laminated micromanufacturing techniques for 3D microneedle arrays. Micromachines.

[37] Caudill C., Perry J.L., Iliadis K., Tessema A.T., Lee B.J., Mecham B.S., Tian S., DeSimone J.M. (2018). Transdermal vaccination via 3D-printed microneedles induces potent humoral and cellular immunity. Proc. Natl. Acad. Sci. USA.

[38] Krieger K.J., Bertollo N., Dangol M., Sheridan J.T., Lowery M.M., O’Cearbhaill E.D. (2019). Simple and customizable method for fabrication of high-aspect ratio microneedle molds using low-cost 3D printing. Microsyst. Nanoeng..

[39] Luzuriaga M.A., Berry D.R., Reagan J.C., Smaldone R.A., Gassensmith J.J. (2018). Biodegradable 3D printed polymer microneedles for transdermal drug delivery. Lab A Chip.

[40] Camović M., Biščević A., Brčić I., Borčak K., Bušatlić S., Ćenanović N., Dedović A., Mulalić A., Osmanlić M., Sirbubalo M., Badnjevic A., Škrbić R., Gurbeta Pokvić L. (2020). Coated 3D Printed PLA Microneedles as Transdermal Drug Delivery Systems. CMBEBIH 2019. CMBEBIH 2019. IFMBE Proceedings.

[41] Park J.H., Allen M.G., Prausnitz M.R. (2006). Polymer microneedles for controlled-release drug delivery. Pharm. Res..

[42] Larrañeta E., Moore J., Vicente-Pérez E.M., González-Vázquez P., Lutton R., Woolfson A.D., Donnelly R.F. (2014). A proposed model membrane and test method for microneedle insertion studies. Int. J. Pharm..

[43] Khanna P., Strom J.A., Malone J.I., Bhansali S. (2008). Microneedle-based automated therapy for diabetes mellitus. J. Diabetes Sci. Technol..

[44] van der Maaden K., Luttge R., Vos P.J., Bouwstra J., Kersten G., Ploemen I. (2015). Microneedle-based drug and vaccine delivery via nanoporous microneedle arrays. Drug Deliv. Transl. Res..

[45] Haq M.I., Smith E., John D.N., Kalavala M., Edwards C., Anstey A., Morrissey A., Birchall J.C. (2009). Clinical administration of microneedles: Skin puncture, pain and sensation. Biomed. Microdevices.

[46] Gill H.S., Denson D.D., Burris B.A., Prausnitz M.R. (2008). Effect of Microneedle Design on Pain in Human Subjects. Clin. J. Pain.

[47] Aggarwal P., Johnston C.R. (2004). Geometrical effects in the mechanical characterizing of microneedle for biomedical applications. Sens. Actuators B Chem..

[48] Davis S.P., Martanto W., Allen M.G., Prausnitz M.R. (2005). Hollow metal microneedles for insulin delivery to diabetic rats. IEEE Trans. Biomed. Eng..

[49] Olatunji O., Das D.B., Garland M.J., Belaid L., Donnelly R.F. (2013). Influence of array interspacing on the force required for successful microneedle skin penetration: Theoretical and practical approaches. J. Pharm. Sci..

[50] Yang M., Zahn J.D. (2004). Microneedle insertion force reduction using vibratory actuation. Biomed. Microdevices.

[51] (2018). Biological Evaluation of Medical Devices—Part 1: Evaluation and Testing Within a Risk Management Process.

